# A Double-Blinded Randomized Controlled Trial: Can Pulsed Electromagnetic Field Therapy Be a Novel Method for Treating Chronic Rhinosinusitis?

**DOI:** 10.3390/medicina60111868

**Published:** 2024-11-14

**Authors:** Nessrien Afify Abed Elrashid, Olfat Ibrahim Ali, Zizi M. Ibrahim, Mohammed A. El Sharkawy, Bodor Bin sheeha, Wafaa Mahmoud Amin

**Affiliations:** 1 Department of Physical Therapy for Surgery, Faculty of Physical Therapy, Cairo University, Giza 12613, Egypt; nessrien.afify@pt.cu.edu.eg; 2 Physical Therapy Program, Batterjee Medical College, Jeddah 21442, Saudi Arabia; olfat_ib@yahoo.com; 3 Department of Basic Science for Physical Therapy, Faculty of Physical Therapy, Cairo University, Giza 12613, Egypt; 4 Department of Rehabilitation Sciences, College of Health and Rehabilitation Sciences, Princess Nourah bint Abdulrahman University, P.O. Box 84428, Riyadh 11671, Saudi Arabia; zmibrahim@pnu.edu.sa (Z.M.I.); bhbinsheeha@pnu.edu.sa (B.B.s.); 5 Department of Otorhinolaryngology, Faculty of Medicine, Al-Azhar University, Cairo 11884, Egypt; mohammed_elsharkawy@azhar.edu.eg; 6 Department of Physical Therapy, College of Nursing and Health Sciences, Jazan University, Jazan 45142, Saudi Arabia; 7 Basic Science Department for Physical Therapy, Faculty of Physical Therapy, Cairo University, Giza 12613, Egypt

**Keywords:** magnetic field, sinusitis, headache, fatigue, sinus opacification, nasal obstruction

## Abstract

*Background and Objectives*: Pulsed electromagnetic field (PEMF) therapy offers a promising approach to treating inflammatory diseases. Its notable anti-inflammatory and antimicrobial effects and enhancement of microcirculation in the nasal mucosa make it a valuable treatment option. Despite its potential, the use of PEMF for chronic rhinosinusitis (CRS) is still in its early stages, with limited exploration of its effectiveness. This study aimed to assess the impact of PEMF on alleviating symptoms such as fatigue, headaches, sinus opacifications, and ostiomeatal complex issues associated with CRS. *Materials and Methods*: Forty-seven patients of both genders with CRS, aged 19 to 40 years, were involved in this study. The participants were randomly assigned to either a magnetic or a control group. The magnetic group underwent a 10 min PEMF session with a 20-gauss magnetic field strength at 7 Hz thrice a week for a month. The control group received the same PEMF application as an inactive device. Before and after the intervention, researchers assessed fatigue levels with a visual analog fatigue scale (VAFS), headache intensity via a numerical pain-rating scale, and the status of sinus opacifications and ostiomeatal complex obstructions by computerized tomography (CT). *Results*: The study findings showed a significant reduction in fatigue and headache scores in the magnetic group compared to the control group (*p* < 0.05). Additionally, there was a notable improvement in sinus opacifications and ostiomeatal complex obstructions among participants who received PEMF therapy. *Conclusions*: PEMF therapy effectively reduces fatigue, headaches, and sinus opacifications in CRS patients, suggesting its potential for inclusion in CRS management guidelines to improve patient outcomes and quality of life. The results of this study indicate that PEMF represents a noninvasive and cost-effective approach for treating adults with mild-to-moderate CRS.

## 1. Introduction

Chronic sinusitis, also known as chronic rhinosinusitis (CRS), is a prolonged inflammatory process affecting the mucosal membranes of the nasal cavities and paranasal sinuses, lasting longer than three months and affecting individuals of all age groups. The most prevalent symptoms are nasal obstruction, facial fullness or pressure, anterior or posterior nasal discharge, and olfactory loss [1,2].

Several factors may contribute to CRS, including viral and bacterial infections, inflammatory reactions associated with allergic rhinitis triggered by environmental irritants such as dust and molds, and exposure to toxins and other noxious substances. Additionally, CRS can result from ciliary dysfunctions and structural abnormalities, such as nasal septum deviations and the formation of nasal polyps. Furthermore, CRS has been linked to other pathological conditions, including otitis media, cystic fibrosis, and asthma [3,4].

The main consequence of CRS is its diverse manifestation, which adversely affects patients’ quality of life (QoL), sleep quality, and daily productivity [5]. Furthermore, missed workdays resulting from visits to physicians or hospitals impose a significant economic burden on patients. The most often disregarded sign of rhinosinusitis is fatigue, described as a state of physical and mental exhaustion which persists despite sufficient rest. Fatigue negatively impacts QoL, even without other related disease processes [6].

The most common secondary headaches are typically related to rhinosinusitis, known as sinus headaches. Chronic rhinosinusitis does not consistently link to headaches, but it does occur in many specific populations—roughly three out of four patients with this syndrome [7].

The diagnosis of CRS relies on specific clinical signs and symptoms, categorized as minor or major. These may not be enough because they can also be found in harmless sinonasal conditions. Moreover, the initial symptoms may not accurately indicate the specific paranasal sinus affected or the extent of the disease’s spread. The European Academy of Allergy and Clinical Immunology recently issued guidelines advocating the incorporation of computerized tomography (CT) scan findings into the CRS diagnostic process, considering CT a gold standard [8].

The traditional sinusitis treatment typically involves pharmacological interventions, such as corticosteroids and antibiotics with potent anti-inflammatory properties. Nevertheless, it is advisable to exercise caution when administering these medications, particularly to children, pregnant women, the elderly, and patients with other medical conditions [2]. Nevertheless, if pharmacological treatment is ineffective, biological therapy and surgical intervention may offer a more productive and cost-effective approach to managing chronic rhinosinusitis [9,10].

A pulsed electromagnetic field (PEMF) is a form of electric energy produced in brief intervals that induces micro-currents, facilitating ion transport within living tissue. This therapy triggers cellular responses in epidermal, fibroblast, leukocyte, and nerve cells. These biological effects typically occur without any discernible side effects or direct contact. The United States Food and Drug Administration (FDA) authorized PEMF therapy in clinical medicine in the 1980s [11].

The literature indicates that PEMF can induce interfering electrical and magnetic fields that can modulate the fundamental frequencies of the electromagnetic fields produced by living tissues [12,13,14,15]. Studies have demonstrated the influence of low-frequency electromagnetic fields on cell proliferation, membrane structure and function, nucleic acids, protein phosphorylation, and adenosine triphosphate (ATP) production [12,16].

A substantial body of research has examined the impact of EMPF on various health outcomes, including pain [17,18], fatigue, headaches, depression, and QoL, in individuals with chronic inflammatory musculoskeletal conditions [19,20,21] and those with neurological disorders [22,23,24,25]. Despite the heterogeneity of studies using EMPF in chronic disease management, more research is needed to investigate its efficacy in addressing chronic upper respiratory tract inflammation, particularly in the context of CRS in adults.

Studies have reported that PEMF exhibits anti-inflammatory and antimicrobial properties while enhancing the microcirculation of the mucosal membrane in the nasal cavity; this is mediated by nitric oxide (NO). PEMF was found to increase NO synthesis, leading to arteriolar vasodilatation, improving oxygenation, and supporting optimal cellular function [26]. It has been demonstrated that CRS causes hypoxia in the epithelial tissue of the sinus mucosa of the airway, resulting in mucus hyperproduction and inflammatory reactions. These, in turn, lead to dysfunction of the airway epithelium, which plays a vital role as a mechanical barrier in innate immunity [3]. PEMF can effectively reduce the lack of oxygen caused by CRS and its associated consequences. PEMF effectively mitigates the oxygen deficiency resulting from CRS and its related consequences. Additionally, studies have demonstrated the antimicrobial effect of PEMF against bacterial infection [27,28], the common cause of CRS. Two previous studies found that PEMF positively affected children’s CRS symptoms after one month of PEMF intervention [29,30]. Furthermore, Kijak and colleagues explored the cumulative impact of electromagnetic fields and LED light radiation on chronic paranasal sinusitis over 30 days. Their treatment approach resulted in complete sinusitis resolution, evaluated through cone beam computed tomography [2].

Although magnetic therapy has been proposed to treat chronic sinusitis, further research is needed to ascertain its efficacy in this context, particularly for adults. Accordingly, this study aimed to examine the effect of pulsed electromagnetic field (PEMF) therapy on chronic rhinosinusitis in adults. This study’s null hypothesis was that PEMF therapy would have no significant effect on fatigue, headaches, sinus opacifications, or ostiomeatal obstruction.

## 2. Materials and Methods

### 2.1. Study Design

Per the Declaration of Helsinki, this double-blind, randomized, controlled trial was conducted between June 2023 and January 2024. The subjects were randomly assigned to one of two groups: the intervention group (the magnetic group), which received active pulsed electromagnetic field (PEMF) treatment, and the control group (placebo), which received inactive PEMF treatment.

### 2.2. Randomization

The randomization process was conducted by an independent assessor who was not involved in data collection. Randomization was achieved using a computer-generated randomization sequence. Group allocation was conducted using sealed, opaque envelopes, ensuring an unbiased selection process. Both participants and assessors were blinded. The Institutional Ethical Committee, Faculty of Physical Therapy at Cairo University, approved this study under the committee’s reference number (P.T.REC/012/003741) on 17 May 2022. It was subsequently registered with a clinical registry number (NCT05865613). We provided all patients with a comprehensive explanation of the study’s objectives and procedures before obtaining informed consent.

### 2.3. Participants

Forty-seven patients of both genders, aged 19 to 40, participated in the current research. The participants were recruited from the Ear, Nose, and Throat (ENT) department of Kasr El-Aini Teaching Hospital in Egypt, and the PEMF intervention was administered by the physiotherapist in the Faculty of Physical Therapy’s outpatient clinic at Cairo University. An ENT consultant assessed all eligible patients to confirm the diagnosis of CRS through a detailed medical history and the independent evaluation of each patient’s SNOT-22 score. All patients were also examined using 0° and 30° rigid endoscopes to confirm the diagnosis further. A CT scan was then performed for each patient, and the Lund–Mackay score was applied to evaluate the scans, with each patient receiving an individual score. The trial admitted subjects with mild-to-moderate symptoms [31], without providing any medication or physical interventions. The study excluded individuals with nasal polyps, a deviated nasal septum, surgery within the last six months, pregnancy or lactation, diabetes mellitus, high blood pressure, cancer, active pulmonary tuberculosis, respiratory disorders, and long-term use of corticosteroids. Furthermore, individuals who had participated in another clinical trial within the previous 30 days, those who were unable to adhere to their treatment plan, and those who had used antihistamines for less than one week, topical corticosteroids, and non-steroidal analgesics for less than two weeks, systemic corticosteroids for less than four weeks, or anti-cholinergic decongestants or other drugs within the previous three days were excluded from the study [32]. We explained the study’s aim and methodology to the participants and obtained written consent from each eligible participant before beginning the study.

### 2.4. Sample Size Computation

**Figure 1:**
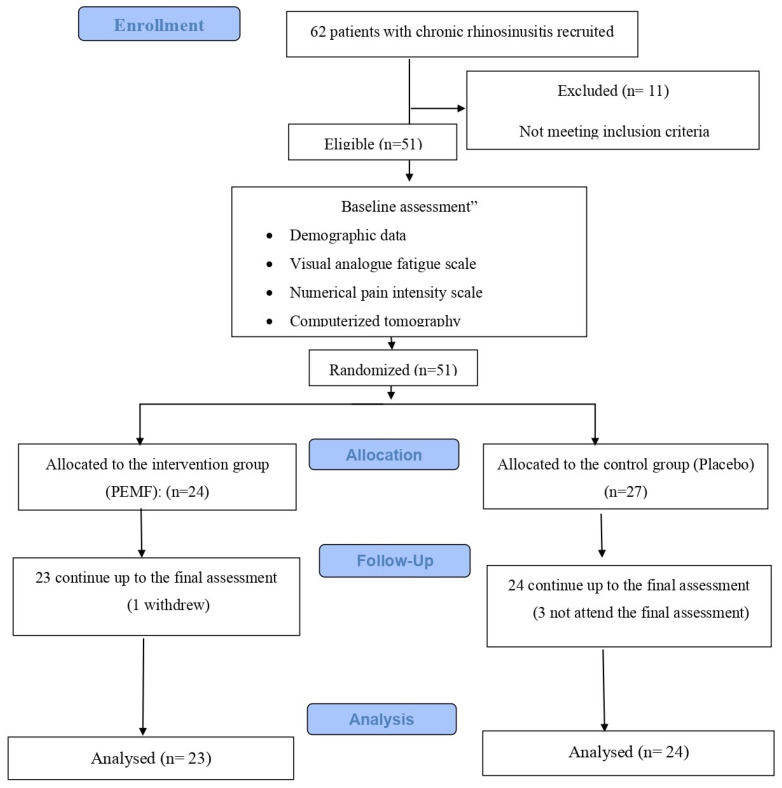
Flowchart diagram.

The G*Power sofware 3.1.9.7 (Universities, Dusseldorf, Germany) was used to estimate the sample size, assuming an effect size of 0.91, a significance level of 0.05, and a power of 0.80; an aggregate sample size of 40 individuals encompassing both groups was necessary. Each group was assigned a sample size of 24 participants to accommodate potential dropout rates after 12 visits, as illustrated in Figure 1 in the flow chart.

### 2.5. Evaluation Procedures

The diagnosis of CRS was made using the criteria outlined in the European Position Paper on Rhinosinusitis and Nasal Polyps (EPOS 2020). According to the EPOS 2020, the diagnosis of CRS necessitates the presence of two or more symptoms, one of which must be nasal blockage, obstruction, congestion, or nasal discharge (anterior or posterior nasal drip). Additional symptoms may include facial pain or pressure and a reduction in or loss of smell that persists for a minimum of 12 weeks. CT scan findings corroborated these symptoms [33]. The Sinonasal Outcome Test 22 (SNOT-22) classified the participants eligible for inclusion in this study as exhibiting mild or moderate symptoms, with an 8–20 score on SNOT-22 for mild symptoms and a score of >20–50 for moderate symptoms [31]. We assessed all the participants’ outcomes both before and after the treatment protocol.

### 2.6. Fatigue Evaluation

The Visual Analog Fatigue Scale (VAFS) is a validated and reliable instrument to assess fatigue severity. The VAFS is a straight line that is 11 cm in length. Both ends of the line bear descriptive labels ranging from “no fatigue” to “very severe fatigue”. We directed patients to mark their level of fatigue on the given line before and after the treatment protocol. The level of fatigue was quantified by the distance between the “no fatigue” endpoint and the patient’s indicated point. A higher value indicated a greater degree of fatigue [34,35].

### 2.7. Evaluation of Headache Intensity

In the context of the headache assessment, we asked patients to assess their headache pain using the eleven-point numerical pain-rating scale, a widely used tool for assessing pain intensity in clinical settings. Researchers have demonstrated the validity and reliability of this method. The scale runs from 0 to 10, with 0 representing no headache and 10 representing a severe headache [36,37].

### 2.8. Computerized Tomography (CT)

**Figure 2:**
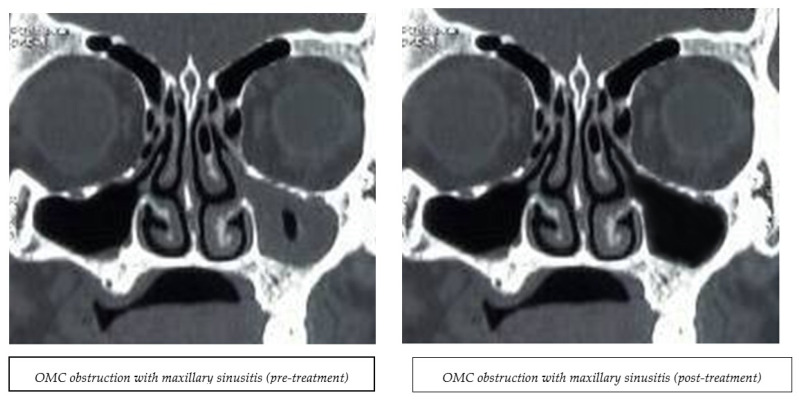
Pre- and post-CT findings after EMFT treatment.

Computerized tomography scanning examinations were conducted at a radiological center before and after the treatment’s completion. The results obtained from the CT films revealed sinus opacification and ostiomeatal complex obstruction. Figure 2 displays the CT findings’ pre- and post-values. The Lund–Mackay score was assessed before and after the study’s completion.

### 2.9. Treatment Procedure

In the magnetic group, the participants received only PEMF therapy using the Automatic PMT Quattro PRO equipment, and no medical treatment was received besides the PEMF. Patients were comfortably positioned within the PEMF device’s solenoid. The physiotherapist initiated the apparatus and selected the optimal program (20 gauss for 10 min, 7 Hz) [35]. The treatment protocol was administered three times per week for one month. The control group underwent the same PEMF procedure, as shown in Figure 2; however, the device remained inactive throughout the same duration and number of sessions, as indicated.

### 2.10. Statistical Analysis

The continuous data exhibited a normal distribution, as evidenced by the Shapiro–Wilk test, box plots, histograms, and mean and standard deviation (SD) calculations. A two-sample *t*-test was employed to assess the demographic data of the participants and the fatigue, headache, SNOT 22, and LMS scales. The fatigue, headache, SNOT 22, and LMS scales were compared using a paired *t*-test. For nominal data, the sinus distribution and ostiomeatal complex were evaluated using McNemar’s and Fisher’s exact tests for comparison within and across groups. The continuous data were presented as the mean and standard deviation, while the nominal data were expressed as numerical values and percentages. The significance level was determined to be less than 0.05 (*p* < 0.05).

## 3. Results

### 3.1. Demographic Data of the Participants

Table 1 demonstrates the demographic data of the participants in both groups. It shows no statistically significant difference between the two groups concerning age, weight, height, BMI, and sex distribution, as the *p*-values are >0.05.

### 3.2. Fatigue Scale

A significant difference was observed between the pre- and post-mean values of the fatigue scale in the study group (*p*-value < 0.001). Before treatment, the mean value of the VAS was 6.72 ± 1.5, while the mean value after treatment was 3.15 ± 1.9, representing a percentage reduction of 53.57%. However, the control group’s pre- and post-VAS mean values showed no significant difference (*p*-value = 0.654). The pre-value was 7.15 ± 1.3, while the post-treatment value was 7.25 ± 0.9.

**Figure 3:**
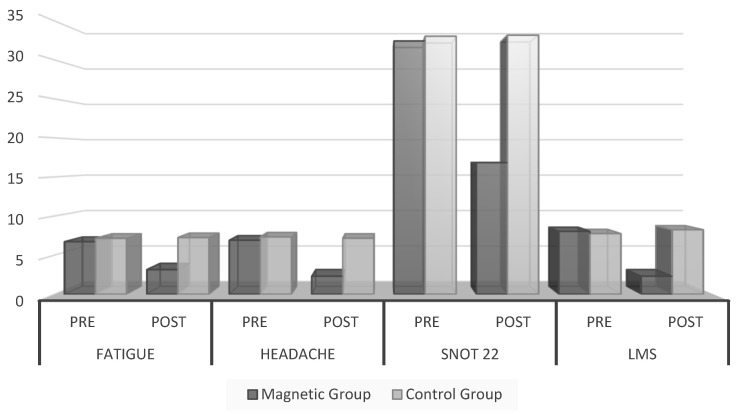
Comparison between groups’ pre- and post-values of fatigue, headache, SNOT 22, and LMS.

A comparison of the groups revealed no statistically significant difference before treatment (*p*-value = 0.297). However, following 12 sessions of treatment, a significant difference emerged, with the magnetic group exhibiting a superior outcome (*p*-value < 0.001). The fatigue scale demonstrated a 53.57% improvement in the magnetic group versus 1.54% in the control group, as illustrated in Table 2 and Figure 3.

### 3.3. Headache Scale

Table 2 and Figure 3 indicate that the data pertain to the headache scale’s values. A statistically significant difference was observed between the magnetic group’s pre- and post-treatment mean numerical pain scale values (*p*-value < 0.001). The mean pre-treatment value was 6.9 ± 1.6, while the mean post-treatment value was 2.3 ± 1.75. However, the control group showed no significant difference between the pre- and post-treatment values (*p*-value = 0.328). The pre-treatment mean value was 7.33 ± 1.2, and the post-treatment mean value was 7.16 ± 1.67.

Regarding the between-group comparisons, we observed no significant difference between the two groups before treatment (*p*-value = 0.328). Conversely, a significant between-group difference was evident post treatment, with the magnetic group exhibiting a superior outcome (*p*-value < 0.001). The headache scale demonstrated a 66.6% improvement in the magnetic group compared to a 2.32% improvement in the control group.

### 3.4. Sinonasal Outcome Test 22 (SNOT-22)

The pre- and post-mean values of SNOT22 are displayed in Table 2 and Figure 3 for the magnetic group. There was a statistically significant difference between the pre- and post-values (*p*-value < 0.001). Conversely, there was no significant difference between the pre-and post-mean values in the control group (*p*-value 0.588). There was no significant difference between both groups pre treatment (*p*-value 0.838); however, there was a significant difference between groups post intervention, favoring the magnetic group (*p*-value < 0.001).

### 3.5. Lund–Mackay Score (LMS)

Table 2 and Figure 3 present the Lund–Mackay score data; the data represent a significant difference between the magnetic group’s pre- and post-mean values (*p*-value < 0.001) but no difference within the control group (*p*-value 0.283). Furthermore, there was no significant difference between the groups before treatment (*p*-value 0.370). After treatment, a significant difference was observed between both groups (*p*-value < 0.001), favoring the magnetic group.

### 3.6. Sinus Opacification and Ostiomeatal Obstruction

The data in Table 3 show no significant difference (*p* > 0.05) between the two groups in the number of opacities in the ethmoid, sphenoid, frontal, and maxillary sinuses before treatment. Following treatment, both groups had no significant difference in the opacification of the right (RT) and left (LT) ethmoid sinuses. However, the PEMF group demonstrated a notable improvement, with 75% of the Rt and Lt opacified ethmoid sinuses clearing up, in contrast to the 0% observed in the control group. A significant proportion of the Rt sphenoid sinuses (66.6%) exhibited complete resolution in the PEMF group compared to the control group. Compared to the control group, the Lt opacified sphenoid sinuses were completely cured in the PEMF group, which showed no disappearance of the opacified sphenoid sinus (*p* = 0.045). In the PEMF group, the resolution of the Rt and Lt opacified frontal sinuses was observed, despite the non-significance level (*p* = 0.243). For the maxillary sinuses, a highly significant difference was present between the groups compared to the control group; all Rt and Lt maxillary sinuses showed complete recovery in the PEMF group (*p* = 0.007).

## 4. Discussion

Clinical studies have investigated the various uses of PEMF therapy in treating orthopedic disorders, neurological diseases, and wound healing. However, there is a paucity of studies assessing magnetic therapy as a potential treatment for adult chronic rhinosinusitis (CRS). Rather than using a representative sample of the adult population, the few case studies conducted have used a small sample size. The current study aimed to assess the efficacy of PEMF therapy in treating CRS in adult patients over a 12-session period. The results showed that PEMF enhanced the measured parameters for treating CRS, leading to the rejection of the null hypothesis. There was a more significant reduction in headache and fatigue levels; the magnetic therapy group experienced a 66.67% decrease in headaches, while the control group only experienced a 2.32% decrease. Similarly, the magnetic therapy group experienced a 53.57% decrease in fatigue, compared to only 1.54% in the control group. Fortunately, the current study’s statistical significance aligned with the clinical significance of the measured outcomes; the mean differences for both headache and fatigue scales were 4.6 and 3.6, respectively, surpassing the minimal clinically important difference (MCID) of 1.41 [38]. The magnetic group reported a difference of 15.7 on the SNOT 22, which aligns with the MCID value of 11.3 [39]. However, the LMS MCID in the literature was inconsistent, ranging from 2 to 6 points, which aligns with the currently reported reduction in LMS [40,41,42].

The CT findings indicated that magnetic therapy application reduced sinus opacification and decreased the opacity of all sinuses. This effect was highly predominant in the left sphenoid sinus, right maxillary sinus, and left maxillary sinus, which might have been due to the variation in sinus volume. This study found that magnetic field therapy effectively cleared up the ostiomeatal blockage for the frontal, maxillary, and ethmoid sinuses. Clearance of the ostiomeatal blockage made fluid flow through the sinuses easier, reducing headaches. It is well known that the inflammatory process in CRS leads to obstruction of the ostiomeatal complex (OMC) and vice versa, resulting in impaired nasal patency due to the concerted bacteria and foreign bodies at the nasal/sinus interface, affecting pH, mucociliary transport and clearance, air temperatures, and flow [43,44].

The results are attributed to PEMF’s effect on cellular activity. PEMF has biophysical properties that penetrate human tissues through magnetic and induced electrical fields. These techniques induce a controlled movement of ions or charged particles within the tissues [45]. PEMF raises the production of calcium (Ca) and nitric oxide (No). It also improves mitochondrial oxygenation and the microcirculation of the mucosal membrane of the nasal cavity. It also helps cells grow back, lowers inflammation in the sinuses, enhances mucociliary function [46], and, finally, ease headaches, tiredness, and OMC blockage. It has been shown that PEMF can reduce mucosal thickness in CRS [30,35] by stimulating lipopolysaccharides (LPSs), which triggers No generation and prolongs the life of free radicals [47,48,49].

This study’s CT results for CRS following PEMF application are consistent with those of a previous study that compared the effects of low-level laser therapy (LLLT) versus PEMF in 30 children with CRS. They randomly assigned the children to two groups (LLLT and PEMF) and treated them for one month (three sessions per week). A CT scanning examination revealed that both groups exhibited comparable improvements in mucosal membrane thickness. The researchers attributed their findings to the analgesic, anti-inflammatory, antimicrobial, and cellular effects of PEMF, noting that these effects were more pronounced on the left-side maxillary sinuses [30]. Previous studies have demonstrated that PEMF can reduce mucosal membrane thickness by promoting tissue inflammation and healing and improving blood circulation [47,50,51,52].

The results of this study are in line with those of Gossili et al. (2015), who demonstrated the efficacy of electromagnetic therapy in treating CRS. The investigators examined the impact of electromagnetic therapy in 52 children with CRS, administering electromagnetic therapy to 20 children in combination with antibiotics (group 1) while the remaining 32 received antibiotics alone (group 2). Two follow-ups evaluated the number, side effects, and recurrence of symptoms. These included paroxysmal nocturnal dyspnea (PND), halitosis or bad breath, stuffy nose, pus discharge from the nose, morning sickness, frontal pain, and maxillary pain. Two weeks after the start of treatment, the initial follow-up revealed no difference between the two groups. However, the group receiving electromagnetic therapy and antibiotic treatment showed a reduction in symptoms in the subsequent follow-up, one month later [29].

Kijak et al. (2022) used cone beam computed tomography (CBCT) in a case study of paranasal sinus inflammation after treatment with an electromagnetic field and light radiation from diodes (LEDs) to examine the anatomical structure of the paranasal sinuses. Their results align with those observed in our study. The case study involved a 39-year-old female with a recurrence history of upper respiratory tract infections that persisted for 2.5 years; a CBCT examination revealed paranasal sinusitis. The patient underwent thirty treatments, with one session per day lasting 20 s. At the end of the sessions, CBCT scanning demonstrated the complete resolution of inflammation in all paranasal sinuses and the restoration of normal sinus pneumatization. This evidence supports the efficacy of electromagnetic therapy for treating sinusitis [2].

This study’s strengths include using low-cost, noninvasive techniques to treat CRS instead of medications and surgical intervention without significant side effects, as reported in the literature [29,30]. Furthermore, this study employed appropriate CT screening to diagnose and assess the targeted treatment. Nevertheless, a potential limitation of this study is the small sample size, frequent visits for the PMEP group, and the lack of assessment of the PEMF’s long-term follow-up effect. Furthermore, our study design did not include a comparison with the standard of care.

## 5. Conclusions

PEMF is a viable treatment for adult CRS of mild-to-moderate severity. It alleviates CRS symptoms, such as headaches and fatigue, and has a favorable safety profile.

## Figures and Tables

**Table 1:** Demographic characteristics of the patients.

	Magnetic Group (*n* = 23) Mean ± SD	Control Group (*n* = 24) Mean ± SD	*p*-Value
Age (years)	30.26 ± 7.27	26.46 ± 6.6	0.067
Sex, no. (F:M) %	17 (73.9%) 6 (26.1%)	14 (58.3%) 10 (41.7%)	0.260
Weight (kg)	71.02 ± 9.7	66.95 ± 9.5	0.153
Height (cm)	170.17 ± 9.87	166.75 ± 10.53	0.252
BMI (Kg/m^2^)	24.5 ± 1.9	23.97 ± 1.5	0.331

*p*-value: significance level; SD: standard deviation; F: female; and M: male.

**Table 2:** Values and results of headache, fatigue scale, SNOT 22, and LMS comparisons in both groups.

Parameters	Magnetic Group (*n* = 23)	Control Group (*n* = 24)	*p*-Value
Fatigue			
Baseline	6.72 ± 1.5	7.15 ± 1.3	0.297
Post	3.15 ± 1.9	7.25 ± 0.9	(<0.001)
MD (95% CI)	3.6 (2.51–4.6)	0.11 (−0.58–0.37)	
% of change	53.57%	1.54%	
*p* value *	(<0.001)	0.654	
Headache			
Baseline	6.9 ± 1.6	7.33 ± 1.2	0.328
Post	2.3 ± 1.75	7.16 ± 1.67	(<0.001)
MD (95% CI)	4.6 (3.65–6.5)	0.17 (−0.18–0.51)	
% of change	66.67%	2.32%	
*p* value *	(<0.001)	0.328	
SNOT22			
Baseline	32.43 ± 9.9	33.04 ± 10.28	0.838
Post	16.83 ± 4.94	33.17 ± 10.29	(<0.001)
MD (95% CI)	15.7 (13.33–17.88)	0.6 (−0.61–0.35)	
% of change	48.1%	0.4%	
*p* value *	(<0.001)	0.588	
LMS			
Baseline	8.04 ± 1.02	7.75 ± 1.19	0.370
Post	2.3 ± 1.7	8.25 ± 2.4	(<0.001)
MD (95% CI)	5.74 (5.01–6.47)	−0.5 (−1.44–0.44)	
% of change	71.4%	6.4%	
*p* value *	(<0.001)	0.283	

*p*-value: between-group significance level; *p*-value *: within-group significance level; MD: mean difference; M: mean; SD: standard deviation; %: percentage; SNOT: sinonasal outcome test; and LMS: Lund–Mackay scale.

**Table 3:** Distribution and comparison of sinus types in both groups.

Sinus Types	Magnetic Therapy Group	Control Group	*p*-Value	Magnetic Therapy Group	Control Group	*p*-Value
	Pre Treatment			Post Treatment		
Rt Ethmoid sinus opacification	4 (17.4.%)	4 (16.7%)	0.947	1 (4.3%)	4 (16.7%)	0.157
Lt Ethmoid sinus opacification	4 (17.4%)	3 (12.5%)	0.681	1 (4.3%)	3 (12.5%)	0.285
Rt Sphenoid sinus opacification	3 (18.8%)	4 (26.7%)	0.685	1 (5%)	4 (26.7%)	
				1 (6.2%)		0.172
Lt Sphenoid sinus opacification	2 (12.5%)	4 (26.7%)	0.394	0 (0%)	4 (26.7%)	0.043
Rt Frontal sinus opacification	1 (4.3%)	1 (4.2%)	0.975	0 (0%)	1 (4.2%)	0.243
Lt Frontal sinus opacification	1 (4.3%)	1 (4.2%)	0.975	0 (0%)	1 (4.2%)	0.243
Rt Maxillary sinus opacification	4(17.4%)	5(20.8%)	0.764	0 (0%)	5 (20.8%)	0.007
Lt Maxillary sinus opacification	5 (21.7%)	5 (20.8%)	0.940	0 (0%)	5 (20.8%)	0.007
Rt Ostiomeatal Obstruction	6 (26.1%)	3 (12.5%)	0.233	0 (0%)	3 (12.5%)	0.040
Lt Ostiomeatal Obstruction	7 (30.4%)	4 (17.4%)	0.297	0 (0%)	4 (17.4%)	0.015

*p*-value: significance level; Rt: Right; and Lt: left.

## Data Availability

The raw data supporting the conclusions of this article will be made available by the authors on request.
